# Unlocking Teacher Job Satisfaction During the COVID-19 Pandemic: a Multi-criteria Satisfaction Analysis

**DOI:** 10.1007/s13132-023-01124-z

**Published:** 2023-02-21

**Authors:** Niki Glaveli, Panagiotis Manolitzas, Eftychia Tsourou, Evangelos Grigoroudis

**Affiliations:** 1 Department of Business Administration, School of Social Sciences, University of the Aegean, Mytilene, Chios, Greece; 2 Department of Tourism, School of Economics, Ionian University, Corfu, Greece; 3 Department of Production Engineering and Management, Technical University of Crete, Chania, Greece

**Keywords:** Job satisfaction, Primary school teachers, MUSA, Strategic decision-making

## Abstract

Research has so far made a rather limited advancement in identifying the contribution of the aspects of the working environment that matter to teachers’ overall job satisfaction (TJS), as well as in providing evidence-based guidelines for improving their working experience. Addressing these deficiencies, the current work uses data related to school working environment facets, i.e., opportunities for self-fulfillment, work intensity/load, salary/income, leadership and collegial relations, and overall TJS, from a sample of 438 public primary school teachers in Greece and applies a multi-criteria decision analysis method (the multi-criteria satisfaction analysis (MUSA)) to identify the contribution of these facets to overall TJS, underline the strong and weak points of TJS based on their importance for teachers and the school’s performance on them, and provide direct action implications for improving primary TJS. The results reveal that all the examined facets are crucial for TJS. Yet, self-fulfillment is the most important contributor to overall TJS and work intensity/load the least significant one. Also, self-fulfillment is the strongest point of TJS that school leaders and policymakers should continue investing on, whilst salary/income is a risk factor that could easily turn into a threat for TJS into the future.

## Introduction

Teacher job satisfaction (TJS) is an administrative psychology variable resulting in many important and far-reaching implications for the school, the individual teacher, the students, the teaching profession, and the society. Regarding schools, research has illustrated that TJS positively relates to higher instructional and school quality, more effective school management, and enhanced school cohesion (Ronfeldt et al., 2013). Also, teachers who feel content with their job work with greater enthusiasm; are less inclined to stress and burn out (Hongying, 2007; Skaalvik & Skaalvik, 2011; Anastasiou & Belios, 2020); are more psychologically healthy; show higher job commitment, enthusiasm, and performance levels (Hongying, 2007, Wolomasi et al., 2019; Baroudi et al., 2020); and demonstrate lower turnover and absenteeism rates (Ingersoll, 2017, European Commission, 2018). Furthermore, the students of satisfied teachers feel better and indicate higher levels of self-esteem and commitment and consequently higher performance and achievement (Spilt et al. 2011; Collie et al. 2012; Kunter et al. 2013; Hardy, 2018). Finally, TJS ameliorates the status of the teaching profession, improves teaching attrition levels (Klassen & Chiu, 2011), and closely relates to the overall quality of life in a society (Gross & Etzioni, 1985; Lawler, 1973; Locke, 1976).

Thus, it is not surprising that a considerable amount of research has been directed on gaining an understanding of the factors that contribute to teachers’ feelings of satisfaction, since if effective measures are adopted to enhance TJS levels, then desirable effects can be maximized, and/or undesirable ones may be minimized. So, a deeper knowledge on TJS determinants related to the school working environment is of particular significance predominantly today, due to the radical changes worldwide in the administration of schools and the expressions of concern about declining satisfaction in the education profession (Toropova et al., 2021). Indeed, the nature of modern teaching work is changing as new approaches to public sector and total quality management appear and, among other, place students in the school context as “customers,” intense interaction with parents is required, and the relations between teachers and the school principal are eroding (Zeichner, 2014; Ingersoll, 2017; Toropova et al., 2021). On top of these during the COVID-19 pandemic, the educational process had to fiercely transfer and adapt to an online teaching and learning format, which is a tremendous challenge, in terms of time, effort, and psychological resources, for all participants (Li & Yu, 2022). This is particularly the case in primary schools where students are much younger, and thus more difficult to adapt, and require the assistance and close monitoring of their parents. Therefore, the COVID-19 pandemic brought about professional role (of teachers and school principals) changes and new requirements for teachers’ digital literacy, higher workload, and stress levels, as well as associated feelings of social alienation. Nonetheless, this change is here to stay, as currently more and more schools tend to adopt virtual education models (i.e., uploading coursework and notes, use of online virtual learning platforms, offering online tutorials) indicating a need for greater attention to understand the nature and causes of TJS that associate with the primary school working environment (Sanchez-Cruzado et al., 2021; Zamora-Antunano et al., 2021).

Nevertheless, despite the steps taken forward, the existing literature on the nature and causes of TJS presents at least three major drawback. First, to the best of our knowledge, only a handful of studies has investigating primary school TJS during the COVID-19 pandemic (Li & Yu, 2022). Second, the relevant literature presents a high level of abstraction and does not underline empirical relationships with direct action implications (i.e., the fulfillment of the promise of evidence-based practice) (Sims, 2020). This requires researchers to go beyond the identification of broad theoretical models to recognize explicit interventions to teachers’ working environment and changes to HRM practices that school leaders and government officials can implement to enhance TJS levels. Third, the extant research on TJS seems not to have placed attention to the idea that boosting JS depends on an in-depth understanding of two aspects: the perceived *importance* of individual work facets for employees and the *organization’s performance* (level of specific facet JS) in providing a satisfactory work environment (Johnson & Holdaway, 1994; Grigoroudis & Siskos, 2010). As McFarlin and Rice (1992) expressed “… only when an individual feels that a job facet is important … will extreme levels of satisfaction or dissatisfaction be experienced. In contrast, when a job facet is perceived to be unimportant, less extreme levels of satisfaction or dissatisfaction will be experienced” (p. 46).

The current study seeks to address the above shortcomings by utilizing a multi-criteria decision analysis (MCDA) method, the multi-criteria satisfaction analysis (MUSA, Siskos et al., 1998; Grigoroudis et al., 2002; Siskos & Grigroudis, 2002; Grigoroudis & Siskos, 2010; Angilella et al., 2014) for data analysis. MUSA deals with building and solving decision and planning problems (e.g., how to improve overall TJS) involving multiple criteria/facets of satisfaction (e.g., workload, relationships with colleagues). Specifically, it uses a preference disaggregation model to produce a set of outputs (e.g., weight pies) (Grigoroudis et al., 2002) assuming that overall satisfaction depends on a number of criteria (Grigoroudis et al., 2002; Grigoroudis & Siskos, 2010) which—in the represent work—refer to the working environment facets of TJS. In total, five aspects of TJS—well recognized in the relevant literature as JS facets related to the school working environment—namely, opportunities provided for self-fulfillment, work intensity/load, salary/income, leadership relations, and collegial relations, were adopted as satisfaction criteria and operationalized by the TJS multi-facet scale proposed by Bolin (2007). Regarding MUSA outputs, two major outputs are produced, presented, and discussed here: the satisfaction criteria weight pie and a SWOT-type diagram. The satisfaction criteria weight pie shows the contribution (weight) of each TJS facet to overall TJS, whilst the SWOT-type diagram underlines the weak and strong points of TJS and provides action directions based both on teachers’ perceptions regarding the performance of their school (evidence-based aspect of analysis) and the importance teachers place on each working environment aspect. This is a key input of the present work since research about JS in education has rather ignored the relationship between importance of JS facets for teachers and overall JS (Johnson & Holdaway, 1994; Judge et al., 2001; Locke, 1976; Small, 2020).

Concluding, the present work by utilizing the MUSA method for data analysis adds to the theory and practice of people management in the school context by providing answers to the following (research) questions: first, what is the contribution of important aspects of the school environment (i.e., opportunities for self-fulfillment, work intensity/load, salary/income, leadership, and collegial relations) to the overall JS of primary school teachers under the shadow of the COVID-19 pandemic? Second, what are the strong and weak points of primary school teachers JS when one considers the importance of working environment aspects for primary school teachers and the schools’ performance (measured as level of specific facet TJS) regarding these aspects? Third, how could school principals and policymakers boost primary school TJS levels based on evidence-based knowledge?

## Literature Review

### Defining TJS

As is in the case of most constructs that belong to the social sciences, one cannot trace a universally accepted definition of JS. Indeed, the theoretical domain of JS is rather wide, as it incorporates the elements that are intrinsic both to the type of the job itself and the working environment which employees perceive as being rewarding, fulfilling, and satisfying or the opposite (Demirtas, 2010). Nevertheless, it could be supported that TJS generally equates with how people feel about their job and its specific aspects. Hoppock (1935) was among the first to adopt this approach, followed by wide referenced authors such as Locke (1976) and Evans (2001) who suggest that JS is an emotional state of mind incorporating feelings determined by the extent to which job expectations are met. However, the evaluation of JS entails a cognitive process as well (Miller et al., 2009). Accordingly, TJS “refers to a teacher’s affective relation to his/her teaching role and is a function of the perceived relationship between what one wants from teaching and what one perceives it is offering to a teacher” (Zembylas & Papanastasiou, 2004, p.359).

### TJS: Measurement Approaches and Instruments

TJS (and JS in general) has been examined and operationalized under two different perspectives, i.e., the global and the faceted ones (Rogelberg, 2007). The first approach is broad and attempts to answer general questions of JS, whilst the second examines facets of JS, e.g., physical environment, relations with supervisor and peers, pay, nature of work, opportunities for development and advancement, recognition, and well-being. Although both approaches can be considered as reasonable ones and have been equally adopted in the JS literature, the global approach may be more appropriate when making time-sensitive decisions, whilst the faceted perspective gives an organization the opportunity to examine and understand how different facets influence one’s attitudes and feelings towards work and thus make specific changes to improve it (Rice et al., 1991; Rogelberg, 2007; Sims, 2020).

To the same direction, TJS has been operationalized both under the global and the faceted perspective, and measurement scales have been developed for measuring JS specifically in the school context or adopted from the wide JS literature (e.g., job descriptiveiIndex (JDI) proposed by Smith et al. (1969), Minnesota Satisfaction Questionnaire (MSQ) developed by Weiss et al. (1967), and the Employee Satisfaction Inventory (ESI) suggested by Koustelios (1991) and Koustelios and Bagiatis (1997)) to serve both approaches.

Focusing on the scales developed specifically for measuring TJS, the Teaching Satisfaction Scale (TSS) put forward by Ho and Au (2006) is an example of a measurement instrument that approaches TJS under the global approach. Some of the most commonly used scales for measuring facets of TJS have been the following: the Purdue Teacher Opinionaire (PTO) (Bentley & Rempel, 1980) which is a 100-item questionnaire that addresses 10 factors of TJS, teacher rapport with the principal, satisfaction with teaching, rapport among teachers, salary, load, curriculum issues, teacher status, community support of education, school facilities and services, and community pressures. Lester (1982) suggested the *Teacher Job Satisfaction Questionnaire* (TJSQ) which incorporates 66 items organized around 9 factors: supervision, colleagues, working conditions, pay, responsibility, work itself, advancement, security, and recognition. Evans and Johnson (1990) came up with the *Teacher Job Satisfaction Scale* that includes 17 items related to freedom on the job, poor working conditions, personal success, salary, recognition, teachers’ needs met, demanding work, adequate equipment, challenging job, job interference with family, principal rewards to teachers, personal satisfaction, decision-making, working conditions, organization of school, and job security. Tarabeh (1995) identified four dimensions (incorporating 25 items) describing TJS: fulfillment of expectations, guidance and assistance from the Ministry of Education, internal conditions of work, and relationship with students and parents. Bolin (2007) developed the *Teacher Job Satisfaction Scale* which incorporates 26 items that span in 5 dimensions: opportunities for self-fulfillment, work intensity/load, salary/income, leadership, and collegial relations. Lastly, other authors have utilized national survey instruments such as the *Teaching and Learning International* *Survey* (TALIS) and the *Teacher Follow-up Survey*. By applying confirmatory factor analyses on 2018 data from the TALIS TJS scale, Zakariya (2020) suggested that TJS is a two-dimensional construct that incorporates two correlated latent constructs: job satisfaction with work environment (JSENV) and job satisfaction with profession (JSPRO). Also, Liu and Ramsey (2008) used 25 items from the *Teacher Follow-up Survey*, and based on factor analysis, they proposed 7 TJS facets labeled as school administration, student interaction, professional development, safety, work conditions, resources, and compensation.

From the presentation of and discussion on the instruments developed to measure TJS, it is obvious that it is a multi-faceted and dynamic construct. Moreover, opportunities for self-fulfillment and development, compensation/rewards, relations with colleagues and the school principal, physical conditions, and workload/work intensity seem to be among the most common facets of TJS that relate to the working environment and are incorporated into the measurement instruments developed to measure TJS.

### TJS Working Environment Facets: Research on Their Importance and Contribution to Overall TJS

The sources of TJS can be classified into the following domains (Sahito & Vaisanen, 2020): (a) factors intrinsic to the job (i.e., working with students, seeing students learn and develop), (b) school-based factors (i.e., school’s working environment) (Skaalivik & Skaalvik. 2011 classify domains a and b as environmental factors), (c) factors extrinsic to the school (i.e., legislation, professional status), (d) psychological (i.e., personality, behavior attitude), and (e) demographic (i.e., age, gender) (Crossman & Harris, 2006). Reviewing the environmental factors (the aim of this study), Bascia and Rottmann (2011) re-established the importance of working conditions in schools for TJS recognizing as crucial factors the following: adequate resources, feasible workload, collegial cooperation, opportunities for professional development, leadership support, and decision-making opportunities. Also, Skaalvick and Skaalvick (2011) studied the impact of environmental factors such as supervisory support, relation with colleagues, and time pressure and found that time pressure was the only factor related weakly to TJS. Johnson et al. (2012) concluded that although a wide range of working conditions matter to teachers, the elements that teachers value the most do not relate to the narrowly conceived working conditions like clean and well-maintained facilities or access to modern instructional technology but rather to leadership and relationships among colleagues. Similarly, Sims (2018, 2020) underlined that working conditions in schools are an important predictor of TJS. More precisely, based on a critical review of the literature, Sims (2020) suggested that teachers’ *progression* (i.e., pay and promotion) and *professional development opportunities, relationships with colleagues and school leadership, workload, and intensity, students’ discipline and feedback* were the elements of teachers’ working environment that matter to teachers the most and contribute to TJS. The results showed strong associations of overall TJS with school leadership, personal development, and scope for career progression. More recently, Nyamubi (2017) found that monetary and non-monetary incentives, community support, fair remuneration packages, opportunities for career development, a well-defined individual appraisal system, timely promotion, workplace conditions, friendship, colleagues’ cooperation, the respect of the community for its members, students’ success and cooperation, and a sense of duty and responsibility were prime factors for TJS. Also, Toropova et al. (2021) identified as important to TJS the following facets of school working conditions, *student discipline*, *leadership support*, *teacher cooperation*, *school resources*, and *workload*, and found that workload, teacher cooperation, and student discipline were the facets that were strongly related to overall TJS.

Nonetheless, research on JS in education has largely ignored the relationship between importance of individual job facets and overall job satisfaction. Indeed, in the school context, Johnson and Holdaway (1994) is probably the only study that examined the above relationship in the school context focusing on school principals and suggesting that initiatives to enhance principals’ satisfaction should better focus on important issues such as involvement in hiring of staff and the performance of students and teachers.

Concluding, from the above studies, it is obvious that an array of working environment facets relates to overall TJS. Specifically, opportunities for development, self-fulfillment, and advancement-progression (e.g., promotion and pay), supervision and relationships with colleagues, working conditions and intensity, resources, and students discipline were underlined as important aspects. These aspects, as expected, are similar to the satisfaction facets that were incorporated in most TJS measurement scales developed under the faceted satisfaction approach. Also, the investigation of the relationship between importance of individual job facets for teachers and JS has received little attention. Moreover, the results regarding the contribution of school working environment facets to TJS are not conclusive.

### The Greek Primary School Environment and TJS Research

Since primary school teachers’ JS is the target variable of the present study, the Greek primary school environment provided the framework for the research. The education system in Greece is still heavily state controlled and centralized leaving few initiatives on the school unit (OECD, 2011). In fact, schools have limited financial resources to manage and little scope for autonomy, decision-making, and participation in human resource management (HRM) decisions related, e.g., to HR planning and selection, promotion, and pay, in addition to narrow responsibility and accountability for students’ learning (OECD, 2011). Whilst more than 80% of school decisions are adopted by the national government, in relation to 35% across OECD countries (OECD, 2018). In general, Greek schools seem to be missing a clear shared vision, a well-defined mission, and a structured quality control and reflection mechanism for their performance.

Recently, the working environment in Greek schools has deteriorated due to “quick fix” solutions that were adopted and the mass reduction of public sector salaries (including public school teachers) following the deep economic crisis that the country faces in the last decade and more. More precisely, primary school teachers experienced a reduction of 25% in their salary which is not expected to increase in the next few years, the teaching hours were extended to 23 per week, and the number of voluntary transfers and detachments has been decreased substantially (Saiti & Papadopoulos, 2015), whilst during the COVID-19 period, teachers had to adjust rapidly to the challenges of online teaching and teleworking. These factors are burdens for achieving high levels of TJS. Regarding the teaching professor, the major motive for being a teacher in Greece—mainly in the past but still relevant to a certain extend—was job security and stability (Anastasiou & Belios, 2020; Saiti & Prokopiadou, 2008).

Research on Greek primary school teachers JS and satisfaction with aspects of working environment is rather limited. The few relevant studies have revealed that Greek teachers were satisfied *with administration*, *the nature of work itself*, *colleagues*, *and supervision*, whereas they are less satisfied with *pay* (salary, benefits, and potential rewards) and *promotional opportunities* (Koustelios, 2001; Saiti & Papadopoulos, 2015).

## Methodology

### Research Context

The public primary school environment in Greece provided the context for our study for at least three reasons. First, as already mentioned in the Introduction section, the work of primary school teachers under the digital educational process imposed during COVID-19 was much more demanding than in secondary schools or universities (Li & Yu, 2022). Second, primary schools are an important link of the educational chain producing more extensive social returns than secondary schools or higher education. Primary schools are the organizations where the values that guarantee socio-cultural identity and continuity are implanted, the fundamentals of human development are set and basic attitudes, and behaviors that will affect a healthy social life are taught (Sfakianaki, 2019). Third, the issue of primary school teachers’ JS has not received much attention, particularly under the shadow of the COVID-19 pandemic (Li & Yu, 2022). Finally, it should be added that private primary schools were excluded as they operate under different administrative and operational condition than public school. For example, more initiatives can be undertaken at the school unit level, they may operate on higher budgets and are more autonomous regarding HRM-related decisions such as promotion and pay. Also, they seem to have higher responsibility and accountability for students’ learning (OECD, 2018).

### Sampling, Data Collection Procedure, and Research Instrument

Regarding the collection procedure, the authors approached the Greek Department of Public Education who, in accordance with its sampling procedures (stratified random sampling), provided a list of the school principals’ email addresses for 2735 schools from urban, semi-urban, and rural areas. It should be noted that the total number of primary schools that were operated in Greece in September 2020 was 4272. So, 64% of the total population of public primary schools was approached in October 2020. Following, the school principals of the 2735 received an email, which included a cover letter and the link for accessing the online questionnaire, and were asked to forward it to their teachers. The cover letter was explaining the aim of the research and assuring for confidentiality and anonymity. The online questionnaire consisted of two sections. The first section included questions designed to determine teachers’ personal characteristics, namely, gender, age, education level, total years of service and years of service with the specific school, specialization, and type of employment. Furthermore, a question on the location of the school was added. The second section incorporated 27 questions measuring facet (26 questions) and overall TJS (1 question).

### Sample Characteristics

In total, 438 valid questionnaires were received. As seen in Table 1, the majority of the teachers were female (75.1%), in their middle adulthood (52.4% were between 31 and 50), and had a rather extensive experience in teaching (44% had a teaching experience of over 6 years). Also, almost 51% of the respondents held a master’s degree, and 56.8% were permanent staff.

**Table 1:** The sample characteristics

**Sample characteristics**	**Percentage**
Biological sex	
Male	24.9
Female	75.1
Age	
22–30	16.9
31–40	29.2
41–50	21.7
51–60	31.7
> 60	0.5
Educational level	
University degree	45.7
Master	50.9
PhD	3.4
Status	
Permanent	56.8
Substitute	43.2
Years of experience	
< 1	15.9
1–5	28.1
6–10	14.4
11–15	25.4
16–20	9.5
21–25	3.5
26–30	2.3
> 30	0.9

### Measurement of Facet Satisfaction and Overall TJS

The instrument developed by Bolin (2007) was adopted for measuring satisfaction with specific working environment elements/facets. The scale incorporates 26 items that span into five facets of TJS labeled as opportunities provided for self-fulfillment (incorporates 7 items that relate to sense of achievement, fulfillment of ideal, exercise of abilities/full potential, respect), work intensity/load (includes 5 items that relate to stress and hygiene), salary/income (includes 5 items that pertain to income and welfare), leadership relations, and collegial relations (these two factors consist of 9 items that concern relationships with the school leader and colleagues). These elements—as underlined earlier in the present paper—have been identified by extant research as the aspects of work environment that matter for teachers and relate to the current developments that characterize the teaching landscape. Also, one (1) item referring to overall JS was added. Respondents were asked to evaluate their working environment aspects, and overall JS using a 5-point Likert-type scale—ranging from 1 = strongly disagree to 5 = strongly agree—was utilized.

### The MUSA Method

The MUSA method was applied for the analysis of our data. MUSA is a multi-criteria decision analysis (MCDA) method that fall in the preference disaggregation approach. This approach offers quantitative measures of satisfaction (i.e., TJS in our work) considering though the qualitative aspect of respondents’ evaluations (Grigoroudis et al., 2002; Grigoroudis & Siskos, 2010). It assumes an additive collective value function $Y^{*}$ and a set of partial satisfaction functions $X_{i}^{*}$. The goal of the method is to attain the maximum consistency between the value function $Y^{*}$ and the respondents’ evaluations $Y$ (see, e.g., Grigoroudis and Siskos (2010), for a detailed presentation of MUSA).

**Fig. 1:**
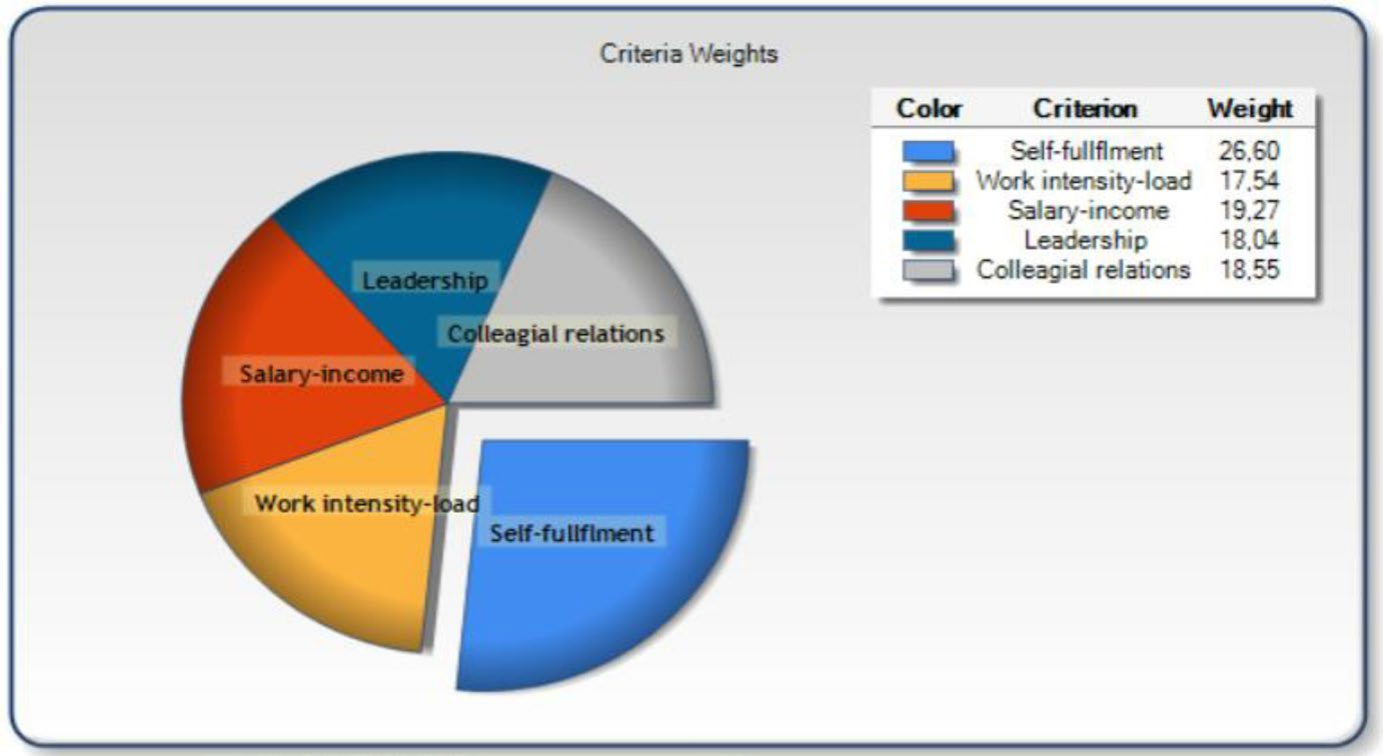
The contribution of the working environment elements to overall TJS

A set of useful outputs like criteria weights (which indicate the criterion’s relative contribution to total/overall satisfaction) and action (SWOT-type) diagrams can be produced by MUSA. These outputs are the focus of the current work and are presented below. Concerning the criteria weights, satisfaction measurement is approached as a multivariate analysis problem given that customers overall (global) satisfaction depends on a set of variables representing work environment dimensions. This set of criteria is denoted as *X* = (*X*1, *X*2, …, *X*n), where a particular criterion *i* is represented as a monotonic variable *X*i. The weights of the criteria show in a range of 0–100% the level of importance of criterion (its contribution) to overall satisfaction (see Fig. 1).

**Fig. 2:**
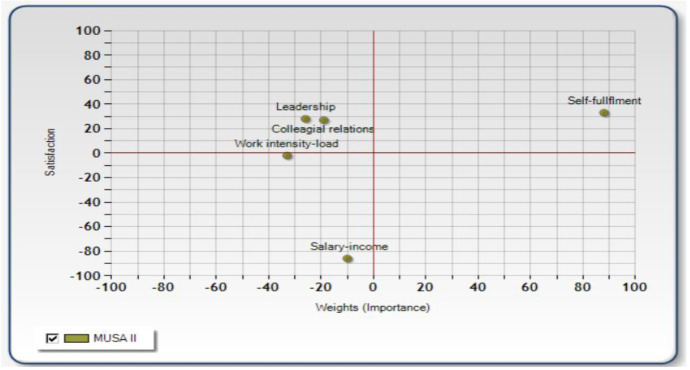
Performance (satisfaction)/importance (SWOT) diagram: the weak and strong points of TJS

Combining weights and average satisfaction indices, an action diagram (SWOT-type analysis) can be developed (Grigoroudis & Siskos, 2010). This diagram (see Fig. 2) shows the strong and weak points of satisfaction organized in four quadrants based on the school’s performance (high/low) regarding the satisfaction criterion (attribute of working environment) and whether each criterion is important for the teachers (high/low). Also, specific directions for actions are provided. More precisely, the satisfaction criteria located in the *low performance (satisfaction)/low importance* (status quo) quadrant require no action for the time being. Aspects that appear in the *high performance(satisfaction)/high importance* (leverage opportunity) quadrant are the criteria that could offer competitive advantage. The items in the *high performance(satisfaction)/low importance* (transfer resources) quadrant require no investment. Lastly, the elements in the *low performance(satisfaction)/high importance* (action opportunity) quadrant call for prompt care by the school leaders, meaning that they represent the weak points of the school environment in terms of TJS.

### Validity and Reliability

Proper attention to any methodological issues that can threaten the validity of an instrument is necessary. Thus, the psychometric properties (discriminant and convergent validity and reliability) of the TJS construct were initially assessed by applying first-order confirmatory factor analysis (CFA) through LISREL software. The outcomes of the CFA are incorporated in Table 1 signifying that the measurement model—based on the provided fit statistics—fits the data well. Cronbach’s alpha coefficient, composite reliability (CR), and the average variance extracted (AVE) were used to assess the internal reliability or consistency of the constructs (Hair et al., 2010). The values of these indices were found to be well above the minimum suggested values of 0.7, 0.7, and 0.5, respectively (see Table 1) (Hair et al., 2010).

Table 2 also displays the convergent validity of the TJS measurement scale which is demonstrated by the fact that all the items were significant (*p* < 0.05) related to their hypothesized factors and that the standardized lambda coefficients were over 0.5 (Schumacker & Lomax, 2004; Steenkamp & van Trijp, 1991). Moreover, discriminant validity was established because the square root of the AVE calculated for each variable was higher than the correlations among the factors (Fornell & Larcker, 1981). Finally, the goodness of fit of the analysis was demonstrated (*p* < 0.05) with the following fit indices: NFI/NNFI, CFI, IFI, and GFI (Bentler, 1990) (see Table 1). All calculated values were greater than 0.9, indicating that the model provides a good fit. In addition, the ratio *X*^2^/df is 2.91 which is below the suggested cutoff point of 3 (Bagozzi & Yi, 1988).

**Table 2:** CFA of the measurement model and reliability indicators

**Factor**	**Item**	**Std. lambda**	**Cronbach’s alpha**	**CR**	**AVE**	**Measurement model**
Opportunities provided for self-fulfillment	OSF1	0.66 0.57	0.86	0.884	0.524	
	OSF2	0.65				
	OSF3	0.74				*X*^2^ = 495.96
	OSF4	0.8				df = 269
	OSF5	0.83				(*p* = 0.0000)
	OFS6	0.78				*X*^2^/df = 1.8
	OSF7					NFI = 0.926
						NNFI = 0.930
Work intensity/load	WIL1 WIL2 WIL3 WIL4	0.68 0.60	0.83	0.833	0.503	CFI = 0.933
	WIL5	0.83				IFI = 0.934
		0.78				GFI = 0.938
		0.63				RMSEA = 0.051
Salary/income	SI1	0.75 0.91	0.77	0.83	0.502	
	SI2	0.56				
	SI3	0.61				
	SI4	0.66				
	SI5					
Leadership relations	LR1 LR2 LR3	0.61 0.75	0.75	0.835	0.509	
	LR4	0.84				
	LR5	0.53				
		0.79				
Collegial relations	CR1	0.71 0.69 0.73	0.79	0.814	0.523	
	CR2 CR3	0.76				
	CR4					

To minimize the risk of CMV, respondents were assured of complete anonymity to minimize any possible doubts or hesitation in completing the questionnaire. Also, Harman’s one factor method and one factor CFA were used to test for the presence of CMV; both tests indicated that CMV was not a serious threat for the data.

## Results

### Overall Job Satisfaction Contributors

Figure 1 illustrates the contribution of each aspect of the working environment on primary school teachers JS. Opportunities for self-fulfillment, i.e., opportunities for achievements and applying knowledge, skills, and full potential, are the variable (criterion) of the school working environment that contributes the most to TJS (weight = 26,60%), followed by salary/income (weight = 19,27%) which refers to absolute and relative income level, subsidies, and welfare. The lower contributor is work intensity/load (weight = 17,54%) that relates to work stress and hygiene conditions.

### Performance/Importance Analysis and Guidelines for Improvement Strategies

Decision-makers in education cannot make decisions for improving overall TJS levels relying only upon knowledge of the importance (weights) of critical aspects of the work environment for overall TJS (knowledge of what is important for them). It is also crucial to take into consideration their perceptions of the school’s performance regarding these attributes (knowledge of what is important to change right now) (Locke, 1976). So, to improve the interpretation of the results presented previously, the action diagram incorporated in Fig. 2 was produced by the MUSA software. The figure depicts the strong and weak points of TJS.

More precisely, based on the positioning of the *self-fulfillment* variable in the high performance (satisfaction)/high importance (leverage opportunity) quadrant, it could be supported that it is the strong aspect of primary teachers’ JS and *can offer competitive advantage.* This means that it is highly valued by teachers’ facet of the working environment, whilst at the same time, the schools perform well regarding this element; so although no immediate action is required for the time being by the school leaders, they should preserve this element of the working environment. *Leadership and collegial relations* appear in the high performance/low importance (transfer resources) quadrant; thus, they are work elements that primary schools perform highly, although they seem to be of rather low importance for teachers. So, no further investment is required. Rather resources invested in these aspects could be transferred. Lastly, *work intensity/load and salary/income* are aspects of the working environment that primary schools’ performance is low; nonetheless, they are also of low importance for teachers (appear in the low performance/low importance or status quo quadrant). Although no immediate action is required, these variables call also for close monitoring by the school leaders since they are risk factors, meaning aspects that could easily be transferred to the low performance/high importance (action opportunity) quadrant.

## Discussion

This work makes several things clearer on TJS. First, it adds to the less well-developed existing literature regarding the contribution of facets of working environment to overall TJS. Second, it underlines the strong and weak points of TJS by taking into consideration the importance of these facets for teachers and school’s performance on them. Third, it provides implicit evidence-based actions for improving TJS. Below, the implications for theory and practice of the present research are presented.

### Theoretical and Methodological Implications

The current research builds on and extends the situational approach to JS by examining the impact of aspects of the school working environment on overall TJS (Judge et al., 2001; Li & Yu, 2022; Toropova et al., 2021), as well as the facet satisfaction theory put forward by Lawler (1973) which recognizes the difference between overall JS and facet satisfaction and defines facet satisfaction as “affective reactions to particular job aspects such as pay, supervision, and opportunities for promotion” (p. 64). Furthermore, we have embraced and provided support for the idea proposed by Johnson and Holdaway (1994) that *overall job satisfaction and facet satisfaction should be investigated as separate variables*. Indeed, our study revealed that specific facets of satisfaction relate to and contribute differently to overall JS. More precisely, the results of MUSA analysis (i.e., the satisfaction criteria weight pie) (Fig. 1) demonstrated that when the contribution of *opportunities for self-fulfillment*, *leadership and collegial relationship*, *work intensity/load*, *and salary/income on TJS* is analyzed simultaneously, *opportunities for self-fulfillment* are the facet (aspect) of the school context that seems to contribute the most to overall TJS. This finding is in line with the results of other studies in the field who suggest that aspects of the job related to sense of achievement, opportunity to exercise one’s full potential, use of competencies, and respectful relationship with students and parents (incorporated as items to the opportunities for self-fulfillment factor in the present work) are essential for achieving higher levels of TJS (Hongying, 2007; Sims, 2020). It seems that a school environment that help teachers satisfy the needs of autonomy, competence, and relatedness (the three pillars of self-fulfillment according to the self-determination theory (Deci & Ryan, 2008; Ryan & Deci, 2002)) can largely contribute to overall TJS (Korthagen & Evelein, 2016). Indeed, self-fulfillment is an important factor for TJS besides an important element in the decision to join and/or leave the profession (Toropova et al., 2021).

Moreover, salary/income was put forward as the second most important contributor to TJS, a result corroborated by previous research (Bolin, 2007). It is interesting to note that in the current study, salary/income was operationalized not only as absolute but also as relative income level, which incorporates an aspect of external reward justice as well. An intrigue result of our study is that it *undervalues the contribution of a feasible workload* to TJS (weight = 17,54%) (Tropova et al., 2021). Nevertheless, this outcome should not be interpreted as unimportance of *work intensity/load* but only as these elements having less weight, compared to the other examined factors, to overall TJS. Research has found that teachers experience an increasing number of work assignments and a more hectic workday, thereby resulting in less time for rest and recovery (Hargreaves, 2003; Lindqvist & Nordänger, 2006; Skaalvik & Skaalvik, 2010)—which may have been expected to result in increasing stress and frustration (Skaalvik & Skaalvik, 2011; Ingersoll, 2017)—this does not seem to be the case for Greek teachers. Perhaps, the fact that an increase in workload/intensity (e.g., extension of working hours) was imposed legally as a fix solution to recover the severe economic crisis may have been accepted as imposed change and inherent aspect of work. It means that high work demands in terms of time and effort and low wages are objective realities for teachers at present (Bolin, 2007) and most probably the “toll” that public primary school teachers have to pay against job security. Also, the survey was undertaken during the COVID-19 lockdown period were teachers may had the flexibility to organize their work in their own pace whilst accommodating needs from home. Also, leadership and collegial relationships have a combined contribution of over 36% to TJS. This is in line with previous research (e.g., Johnson et al., 2012), which confirms that working conditions of a social nature are more important to teachers than material ones (Sims, 2018), can boost TJS (Reeves et al., 2017; Skaalvik & Skaalvik, 2011), and shield against teachers turnover.

In addition, with this work, it was tried to answer calls for research in the TJS literature that *improves the level of abstraction* and *recognizes empirical relationships with direct action implications* (Sims, 2020). To do so, MUSA an MCDA method was applied for data analysis for the first time in TJS research. So, TJS was approached as a managerial problem, i.e., how to improve overall TJS involving multiple criteria/facets that could contribute to its solution. What is most important is that MUSA analysis helped also address the voices of authors in the field who ask researchers to *consider*—*besides teachers’ perceptions regarding facet satisfaction*—*the importance of individual job facets for teachers* as well (Johnson & Holdaway, 1994; Small, 2020) when designing interventions to ameliorate the level of TJS and maximize desirable effects whilst minimizing undesirable ones.

Johnson and Holdaway (1994) emphasize that job satisfaction ratings alone should not be relied upon to assess the importance of facets for TJS and advice researchers “to investigate levels of overall and facet satisfaction as well as perceptions of importance in studies of educators' job satisfaction” (p. 31). Accepting this view, the authors have practically combined the two features of importance proposed by Locke (1976)—importance in the frame of a person’s value hierarchy and importance to change immediately—to evaluate the strong and weak points of TJS. Specifically, the SWOT-type analysis that was performed was based on the importance of the examined facets of the working environment for teachers and their perceptions on the school’s performance regarding these aspects providing thus specific (rather than abstract) theoretical conclusions and concrete directions for practice (Sims, 2020). The outcomes of the SWOT analysis showed that *opportunities for self-fulfillment* are the strong point of satisfaction in Greek schools (high performance/high importance element of TJS) that needs to be preserved, whilst *work intensity/load and (and particularly) salary/income* are risk factors (low performance/low importance element of TJS) since they could easily move to the low performance/high importance (status quo) quadrant. It is interesting to note that (to the best of our knowledge) the only other research that has taken into consideration both satisfaction and importance for examining job satisfaction in the school context was conducted almost 30 years ago (Johnson & Holdaway, 1994) and also demonstrated that salary, fringe benefits in the contract, and number of hours the principal is required to work were the items of satisfaction with the lowest satisfaction and importance level, whilst with respectful relationships with teachers and students are elements of the school environment that principals valued the most. Thus, it could be supported that school teachers seem to choose and enjoy practicing teaching mainly for reasons related to the satisfaction of self-fulfillment and social needs. So, in schools that meet the self-fulfillment and social needs of their teachers, teachers can be genuine, can implement their own ideas, and make choices, and thus, they can gain advantages such as psychological health and growth, intrinsic motivation, well-being, optimal functioning, and self-actualization (Ryan & Deci, 2002; Baroudi et al., 2020) which may reflect to the school’s overall performance as well.

### Implications for Practice

Research evidence suggests that the school working environment is deteriorating resulting to low levels of TJS, whilst the poor working conditions of a school challenge the status of the teaching profession and hinder the recruitment of new teachers (Toropova et al., 2021). Thus, it is important to provide relevant stakeholders with concrete evidence-based actionable insights and directions on which aspects/facets of the working environment managerial actions should be prioritized (due to resources limitations) to improve TJS. The present research achieves that by producing—through MUSA—findings based on both facet and overall satisfaction and on the significance of these facets for teachers and thus offers valuable information regarding the altering experiences and attitudes of teachers. As a result, our work aids in notifying policy making in ways that could improve teachers’ working lives and consequently the quality of education offered (Johnson & Holdaway, 1994).

From our research findings, it is apparent that for school leaders to bolster their teachers’ JS, they should set as priority to create a working environment that provides teachers with *opportunities for self-fulfillment*, an element that Greek teachers seem to be happy with (schools’ performance on self-fulfillment is high) and at the same time attach high importance to. So schools, who score high on this element, have “competitive advantage” and are expected to provide higher quality of service. As it is clear from the items used to operationalize this facet of TJS, self-fulfillment needs relate to competence (e.g., feel competent in managing the classroom), relatedness (e.g., close contact and a positive/respectful connection with pupils and parents), and autonomy (e.g., be authentic, apply own ideas and choices, and develop accordingly) (Bolin, 2007; Korthagen & Evelein, 2016; Richter et al., 2022). So, school principals who lead in a *transformational and participative* style, such as carrying for the needs, ideas, and interests of their teachers, offering intellectual stimuli to them, raising teachers’ expectations and motivation to devote, and putting extra efforts, are expected to create an environment that can accommodate their teachers’ self-fulfillment needs (Sims, 2020). Also, facilitating primary school teachers to engage in training activities with the scope of developing the adequate knowledge and skills to master their classes and stimulate their students for engaging in learning can be promising for enhancing TJS (Deci & Ryan, 2008; Li & Yu, 2022). Moreover, assisting teachers work on specific and clear learning goals that can be attained with rather little and understandable steps, each of which with a high possibility of accomplishment opportunities, is as well a way towards improving TJS (Maas et al., 2022). Certainly, opportunities for self-fulfillment can be also indirectly affected by governmental policies such as providing the resources and the necessary time off for training, in addition to applying (particularly soft) TQM policies and practices in the primary Greek education system.

In addition, the social environment of the school (collegial and principal relations) seems to contribute to overall TJS level and be quite important for teachers. So, besides adopting a transformational and participative leadership style, Greek primary school principals could also invest (but not for the time being as a priority, since currently schools perform rather high on this issue) on modeling collaborative behaviors and providing specific and protected time slots during working hours to facilitate teachers work in teams which hold most promising for improving relations and consequently TJS. This suggests that leaders should concentrate on setting clear directions and vision for their school whilst giving opportunities to teachers to contribute to decision-making and hold each other up (Maas et al., 2022). For example, school principals could emphasize clear communication and dialog with their teachers to assure that they can express their views and comprehend the underlying principles behind important decisions (Sims, 2020).

Additionally, work intensity/load and salary/income are low performance–and for the time being low importance—elements of TJS. Nevertheless, particularly salary/income can be easily transferred to the low performance/high importance (action opportunity) quadrant (as seen in Fig. 2) and thus represents a risk factor for TJS. Consequently, these aspects need to be closely monitored by the school leaders and the Greek central government that is actually the decision-maker regarding both issues. Actions that could be considered for alleviating work intensity/load (although again–based on our findings—not an immediate priority for actions to be taken) would probably be to give teachers an assignment within their main subject area (Grissom et al., 2015) and provide suitable working conditions (Sims, 2020). It is a fact that in some cases a warm, clean, and safe working/classroom environment is not taken for granted (particularly for schools located in urban areas) due to financial resource shortages.

Finally, school leaders and governmental officials are advised to periodically review, with the use of MCDA methods like MUSA and changes on schools’ performance regarding teachers’ satisfaction with aspects of their working environment, as well as on the importance they attach to them, since only then optimal decisions about enhancing TJS can be made (Johnson & Holdaway, 1994).

### Limitations and Future Research

This study suffers from several limitations, and thus, results should be interpreted with caution. First, despite the fact that the instrument used to measure TJS incorporated quite a few aspects of the school working environment, some of those included in the vast body of literature in the field (e.g., school culture) could not be considered. Second, the sample size threatens the generalisability of our results. Thus, an analysis of TJS and/or a research with a larger sample and a longitudinal component would be important. So, future research should focus on acquiring country level and/or panel data following schools over time. The latest would allow investigation of the relationship between working conditions and TJS over time. Also, teachers may have different aspirations and needs according to, e.g., their gender, career stage, professional growth, or personal needs (Crossman & Harris, 2006; Tropova et al., 2021). So, an analysis of data based on demographic or personal criteria could provide more in-depth knowledge on TJS, providing thus opportunities for more robust decisions regarding the actions that could be implemented to enhance TJS of these different groups of teachers.

## Conclusion

Despite limitations, the present study makes several contributions to the theory and practice of primary school management. To the best of our knowledge, it is the first study that applies MUSA (an MCDA method) to approach TJS, dealing with it as a managerial problem (i.e., how to improve overall TJS level) to be solved considering multiple criteria/facets of the school’s context (TJS facets) that could contribute to its solution, i.e., opportunities provided for self-fulfillment, work intensity/load, salary/income, leadership relations, and collegial relations. Furthermore, it identifies opportunities for fulfillment and the social environment of schools as the aspects of working environment which robustly contribute to JS of primary school teachers in Greece. Also, our study provides concrete evidence-based directions to school leaders and governmental officers who want to improve TJS level, based not only on teachers’ perceptions regarding their school’s performance but also on the importance they attach to each satisfaction criterion (JS facet), assisting thus optimal decision-making. Opportunities for fulfillment seem to be the strong point of TJS in Greek primary schools, whilst salary/income appears to be its weakest point that calls for close monitoring.

## Data Availability

The data presented in this study are available on request from the corresponding author.

## References

[CR1] Anastasiou, S., & Belios, E. (2020). Effect of age on job satisfaction and emotional exhaustion of primary school teachers in Greece. *European Journal of Investigation in Health Psychology and Education,**10*(2), 644–655. 10.3390/ejihpe10020047 34542525 10.3390/ejihpe10020047 PMC8314280

[CR2] Angilella, S., Corrente, S., Greco, S., & Słowiński, R. (2014). MUSA-INT: Multicriteria customer satisfaction analysis with interacting criteria. *Omega,**42*(1), 189–200. 10.1016/j.omega.2013.05.006

[CR3] Bagozzi, R., & Yi, Y. (1988). On the evaluation of structural equation models. *Journal of the Academy of Marketing Sciences,**16*(1), 74–94. 10.1007/BF02723327

[CR4] Baroudi, S., Tamim, R., & Hojeij, Z. (2020). A quantitative investigation of intrinsic and extrinsic factors influencing teachers’ job satisfaction in Lebanon, *Leadership and Policy in Schools*, *21*(2), 127–146. 10.1080/15700763.2020.1734210

[CR5] Bascia, N., & Rottmann, C. (2011). What’s so important about teachers’ working conditions? The fatal flaw in North American educational reform. *Journal of Education Policy,**26*(6), 787–802. 10.1080/02680939.2010.543156

[CR6] Bentler, P. M. (1990). Comparative fit indexes in structural models. *Psychological Bulletin,**107*(2), 238–246. 10.1037/0033-2909.107.2.238 2320703 10.1037/0033-2909.107.2.238

[CR7] Bentley, R., & Rempel, A. (1980). *Manual for the Purdue teacher opinionaire*. West Lafayette, Ind.: Purdue Research Foundation: Distributed by the University Book Store.

[CR8] Bolin, F. (2007). A study of teacher job satisfaction and factors that influence it. *Chinese Education and Society,**40*(5), 47–64. 10.2753/CED1061-1932400506

[CR9] Collie, R. J., Shapka, J. D., & Perry, N. E. (2012). School climate and social-emotional learning: Predicting teacher stress, job satisfaction, and teaching efficacy. *Journal of Educational Psychology,**104*(4), 1189–1204. 10.1037/a0029356

[CR10] Crossman, A., & Harris, P. (2006). Job satisfaction of secondary school teachers. *Educational Management Administration and Leadership*, *34*(1), 29–46. 10.1177/2F1741143206059538

[CR11] Deci, E. L., & Ryan, R. M. (2008). Self-determination theory: A macrotheory of human motivation, 182– development, and health. *Canadian Psychology,**49*(3), 182–185. 10.1037/a0012801

[CR12] Demirtas, Z. (2010). Teachers’ job satisfaction levels. *Procedia - Social and Behavioral Sciences,**9*, 1069–1073. 10.1016/j.sbspro.2010.12.287

[CR13] European Commission/EACEA/Eurydice. (2018). *Teaching careers in Europe: Access, progression and support*. Eurydice Report. Luxembourg: Publications Office of the European Union.

[CR14] Evans, L. (2001) Delving deeper into morale, job satisfaction and motivation among education professionals: Re-examining the leadership dimension. *Educational Management and Administration*, *29*(3), 291–306. 10.1177/2F0263211X010293004

[CR15] Evans, V., & Johnson, D. J. (1990). The relationship of principals’ leadership behavior and teachers’ job satisfaction and job-related stress. *Journal of Instructional Psychology,**17*(1), 11–18.

[CR16] Fornell, C., & Larcker, D. F. (1981). Evaluating structural equation models with unobservable variables and measurement error. *Journal of Marketing Research,**18*(1), 39–50. 10.2307/3151312

[CR17] Grigoroudis, E., & Siskos, Y. (2010). MUSA: Multicriteria satisfaction analysis in: E. Grigoroudis and Y. Siskos (Eds) Customer Satisfaction. *International Series in Operations Research and Management Science, 139, *(pp. 91–121). Springer, Boston, MA. 10.1007/978-1-4419-1640-2_4

[CR18] Grigoroudis, E., Politis, Y., & Siskos, Y. (2002). Satisfaction benchmarking and customer classification: An application to the branches of a banking organization. *International Transactions in Operational Research,**9*(5), 599–618. 10.1111/1475-3995.00376

[CR19] Grissom, J. A., Kalogrides, D., & Loeb, S. (2015). The micropolitics of educational inequality: The teacher–student assignments. *Peabody Journal of Education,**90*(5), 601–614. 10.1080/0161956X.2015.1087768

[CR20] Gross, E., & Etzioni, A. (1985). *Organizations in society*. Pearson College Div.

[CR21] Hair Jr., J. F., Black, W. C., Babin, B. J., & Anderson, R. E. (2010). *Multivariate data analysis: A global perspective*. 7th Edition, Pearson Education, Upper Saddle River.

[CR22] Hardy, I. (2018). Governing teacher learning: Understanding teachers’ compliance with and critique of standardization. *Journal of Education Policy,**33*(1), 1–22. 10.1080/02680939.2017.1325517

[CR23] Hargreaves, A. (2003). *Teaching in the knowledge society: Education in the age of insecurity*. Teachers College Press.

[CR24] Ho, C.-L., & Au, W.-T. (2006). Teaching satisfaction scale: Measuring job satisfaction of teachers. *Educational and Psychological Measurement,**66*(1), 172–185. 10.1177/0013164405278573

[CR25] Hongying, S. (2007). Literature review of teacher job satisfaction. *Chinese Education and Society,**40*(5), 11–16. 10.2753/CED1061-1932400502

[CR26] Hoppock, R. (1935). *Job satisfaction*. Harper.

[CR27] Ingersoll, R. (2017). Misdiagnosing America’s teacher quality problem. In G. K. LeTendre & M. Akiba (Eds.), *International handbook of teacher quality and policy* (pp. 79–96). Routledge.

[CR28] Johnson, N. A., & Holdaway, E. A. (1994). Facet importance and the job satisfaction of school principals. *British Educational Research Journal,**20*(1), 17–33. 10.1080/0141192940200103

[CR29] Johnson, S. M., Kraft, M. A., & Papay, J. P. (2012). How context matters in high-need schools: The effects of teachers’ working conditions on their professional satisfaction and their students’ achievement. *Teachers College Record,**114*(10), 1–39.24013958

[CR30] Judge, T. A., Thoresen, C. J., Bono, J. E., & Patton, G. K. (2001). The job satisfaction-job performance relationship: A qualitative and quantitative review. *Psychological Bulletin,**127*(3), 376–407. 10.1037/0033-2909.127.3.376 11393302 10.1037/0033-2909.127.3.376

[CR31] Klassen, R. M., & Chiu, M. M. (2011). The occupational commitment and intention to quit of practicing and pre-service teachers: Influence of self-efficacy, job stress, and teaching context. *Contemporary Educational Psychology,**36*(2), 114–129. 10.1016/j.cedpsych.2011.01.002

[CR32] Korthagen, F. A. J., & Evelein, F. G. (2016). Relations between student teachers’ basic needs fulfillment and their teaching behavior. *Teaching and Teacher Education,**60*, 234–244. 10.1016/j.tate.2016.08.021

[CR33] Koustelios, A. (1991). *The relationships between organizational cultures and job satisfaction in three selected industries in Greece*. PhD Dissertation. United Kingdom: University of Manchester, Faculty of Education.

[CR34] Koustelios, A. D. (2001). Personal characteristics and job satisfaction of Greek teachers. *The International Journal of Educational Management,**15*(7), 354–358. 10.1108/EUM0000000005931

[CR35] Koustelios, A., & Bagiatis, K. (1997). The Employee Satisfactory Inventory (ESI): Development of a scale to measure satisfaction of Greek employees. *Educational and Psychological Measurement*, *57*(3), 469–476. 10.1177/2F0013164497057003008

[CR36] Kunter, M., Kleickmann, Th., Klusmann, U., & Richter, D. (2013). The development of teachers’ professional competence in: M. Kunter, J. Baumert, W. Blum, U. Klusmann, S. Krauss and M. Neubrand (Eds) *Cognitive Activation* in *the Mathematics Classroom and Professional Competence of Teachers*. Vol. Eight: *Mathematics Teacher Education* (pp. 63–77). Springer, Boston, MA. 10.1007/978-1-4614-5149-5_4

[CR37] Lawler, E. E. (1973). *Motivation in work organisations*. Monterey, CA: Brooks/Cole.

[CR38] Lester, P. E. (1982). *Teacher job satisfaction questionnaire*. Long Island University.

[CR39] Li, M., & Yu, Z. (2022). Teachers’ satisfaction, role, and digital literacy during the COVID-19 pandemic. *Sustainability*, *14*, 1121. 10.3390/su14031121

[CR40] Lindqvist, P., & Nordänger, U. K. (2006). Who dares to disconnect in the age of uncertainty? Teachers’ recesses and ‘off-the-clock’ work. *Teachers and Teaching Theory and Practice,**12*(6), 623–637. 10.1080/13540600601029637

[CR41] Liu, X. S., & Ramsey, J. (2008). Teachers’ job satisfaction: Analyses of the teacher follow-up survey in the United States for 2000–2001. *Teaching and Teacher Education,**24*(5), 1173–1184. 10.1016/j.tate.2006.11.010

[CR42] Locke, E. A. (1976). The nature and causes of job satisfaction. In M. D. Dunnette (Ed.), *Handbook of industrial and organizational psychology* (pp. 1297–1343). Rand McNally.

[CR43] Maas, J., Schoch, S., Scholz, U., Rackow, P., Schuler, J., Wegner, M., & Keller, R. (2022). School principals’ social support and teachers’ basic need satisfaction: The mediating role of job demands and job resources. *Social Psychology of Education, Published Online.*10.1007/s11218-022-09730-6 10.1007/s11218-022-09730-6 PMC9771846 36570365

[CR44] McFarlin, D. B., & Rice, R. W. (1992). The role of facet importance as a moderator in job satisfaction processes. *Journal of Organizational Behavior,**13*(1), 41–54. 10.1002/job.4030130105

[CR45] Miller, H. A., Mire, S., & Kim, B. (2009). Predictors of job satisfaction among police officers: Does personality matter? *Journal of Criminal Justice,**37*(5), 419–426. 10.1016/j.jcrimjus.2009.07.001

[CR46] Nyamubi, G. J. (2017) Determinants of secondary school teachers’ job satisfaction in Tanzania. *Education Research International, *7282614. 10.1155/2017/

[CR47] OECD. (2011). *Education policy advice for Greece: Strong Performers and successful reformers in education,* OECD Publishing, available at: https://www.oecd-ilibrary.org/education/education-policy-advice-for-greece_9789264119581-en. Retrieved 23 January 2021.

[CR48] OECD. (2018). *Education for a bright future in Greece, reviews of national policies for education*. Paris: OECD Publishing, available at: https://www.oecd-ilibrary.org/education/education-for-a-bright-future-in-greece_9789264298750-en. Retrieved 23 January 2021.

[CR49] Reeves, P. M., Pun, W. H., & Chung, K. S. (2017). Influence of teacher collaboration on job satisfaction and student achievement. *Teaching and Teacher Education*, *67*, 227–236. 10.1016/j.tate.2017.06.016

[CR50] Rice, R. W., Gentile, D. A., and McFarlin, D. B. (1991). Facet importance and job satisfaction. *Journal of Applied Psychology*,76(1), 31–39. 10.1037//0021-9010.76.1.31

[CR51] Richter, E., Lucksna, C., Redding, C., & Richter, D. (2022). Retention intention and job satisfaction of alternatively certified teachers in their first year of teaching. *Teacher and Teaching Education. Available Online.*10.1016/j.tate.2022.103704

[CR52] Rogelberg, S. G. (Ed.). (2007). *Encyclopedia of industrial and organizational psychology*: Volume 1. Thousand Oaks, CA: SAGE.

[CR53] Ronfeldt, M., Loeb, S, & Wyckoff, J. (2013). How teacher turnover harms student achievement. *American Educational Research Journal*, *50*(1), 4–36. 10.3102/2F0002831212463813

[CR54] Ryan, R. M., & Deci, E. L. (2002). Overview of self-determination theory: An organismic-dialectical perspective. In E. L. Deci & R. M. Ryan (Eds.), *Handbook of self-determination research* (pp. 3–33). University of Rochester Press.

[CR55] Sahito, Z., & Vaisanen, P. (2020). Literature review on teachers’ job satisfaction in developing countries: Recommendations and solutions for the enhancement of the job. *Review of Education,**8*(1), 3–34. 10.1002/rev3.3159

[CR56] Saiti, A., & Papadopoulos, Y. (2015). School teachers’ job satisfaction and personal characteristics: A quantitative research study in Greece. *International Journal of Educational Management,**29*(1), 73–97. 10.1108/IJEM-05-2013-0081

[CR57] Saiti, A., & Prokopiadou, G. (2008). The demand for higher education in Greece. *Journal of Further and Higher Education,**32*(3), 285–296. 10.1080/03098770802221080

[CR58] Sanchez-Cruzado, C., Campion, R. S., & Sanchez-Compa, M. T. (2021). Teacher digital literacy: The indisputable challenge after COVID-19. *Sustainability,**13*(4), 1858. 10.3390/su13041858

[CR59] Schumacker, R. E., & Lomax, R. G. (2004). *A beginner’s guide to structural equation modeling* (2nd ed.). Lawrence Erlbaum Associates Publishers.

[CR60] Sfakianaki, E. (2019). A measurement instrument for implementing total quality management in Greek primary and secondary education. *International Journal of Educational Management,**33*(5), 1065–1081.

[CR61] Sims, S. (2018). *Essays on the recruitment and retention of teachers*. Ph.D. thesis, available at: http://discovery.ucl.ac.uk/10053430/. Retrieved 25 January 2021.

[CR62] Sims, S. (2020). Modelling the relationships between teacher working conditions, job satisfaction and workplace mobility. *British Educational Research Journal,**46*(2), 301–320. 10.1002/berj.3578

[CR63] Siskos, Y., & Grigoroudis, E. (2002). Measuring customer satisfaction for various services using multicriteria analysis in: Bouyssou D., Jacquet-Lagrèze E., Perny P., Słowiński R., Vanderpooten D., Vincke P. (Eds) *Aiding decisions with multiple criteria. International series in operations research and management scienc*e. Vol. Forty-four: *International Series in Operations Research and Management Science* (pp. 457–482). Springer, Boston, MA. 10.1007/978-1-4615-0843-4_20

[CR64] Siskos, Y., Grigoroudis, E., Zopounidis, C., & Saurais, O. (1998). Measuring customer satisfaction using a collective preference disaggregation model. *Journal of Global Optimization,**12*(2), 175–195. 10.1023/A:1008262411587

[CR65] Skaalvik, E. M., & Skaalvik, S. (2011). Teacher job satisfaction and motivation to leave the teaching profession: Relations with school context, feeling of belonging, and emotional exhaustion. *Teaching and Teacher Education,**27*(6), 1029–1038. 10.1016/j.tate.2011.04.001

[CR66] Skaalvik, E. M., & Skaalvik, S. (2010). Teacher self-efficacy and teacher burnout: A study of relations. *Teaching and Teacher Education,**26*(4), 1059–1069. 10.1016/j.tate.2009.11.001

[CR67] Small, C. (2020). A comparison of public and private school teachers’ job satisfaction when controlling for policy perspectives, individual, and workplace characteristics. Doctor of Education in Educational Leadership for Learning Dissertations. 24, available at https://digitalcommons.kennesaw.edu/educleaddoc_etd/24. Retrieved 23 January 2021.

[CR68] Smith, P. C., Kendall, L., & Hulin, C. L. (1969). *The measurement of satisfaction in work and retirement: A strategy for the study of attitudes*. Rand McNally.

[CR69] Spilt, J. L., Koomen, H. M. Y., & Thijs, J. T. (2011). Teacher wellbeing: The importance of teacher - student relationships. *Educational Psychology Review,**23*, 457–477. 10.1007/s10648-011-9170-y

[CR70] Steenkamp, J.-B.E.M., & Van Trijp, H. C. M. (1991). The use of LISREL in validating marketing constructs. *International Journal of Research in Marketing,**8*(4), 283–299. 10.1016/0167-8116(91)90027-5

[CR71] Tarabeh, H. (1995). *Principals’ and teachers’ job satisfaction as a function of the gap between principal’s perception and teacher’s perception of the principal’s role*. Unpublished Master’s thesis. Israel: University of Haifa (Hebrew)

[CR72] Toropova, A., Myrberg, E., & Johansson, S. (2021). Teacher job satisfaction: The importance of school working conditions and teacher characteristics. *Educational Review,**73*(1), 71–97. 10.1080/00131911.2019.1705247

[CR73] Weiss, D. J., Dawis, R. V., England, G. W., & Lofquist, L. H. (1967). Manual for the Minnesota Satisfaction Questionnaire. Vol. 22, Minnesota Studies in Vocational Rehabilitation, Minneapolis: University of Minnesota, Industrial Relations Center, available at: https://vpr.psych.umn.edu/sites/vpr.umn.edu/files/files/monograph_xxii_-_manual_for_the_mn_satisfaction_questionnaire.pdf. Retrieved 25 January 2021

[CR74] Wolomasi, A. K., Asaloei, S. I., & Werang. B. R. (2019). Job Satisfaction and performance of elementary school teachers. *International Journal of Evaluation and Research in Education*, *8*(4), 575–580. 10.11591/ijere.v8i4.20264

[CR75] Zakariya, Y. F. (2020). Investigating some construct validity threats to TALIS 2018 Teacher Job Satisfaction Scale: Implications for social science researchers and practitioners. *Social Sciences,**9*(4), 38. 10.3390/socsci9040038

[CR76] Zamora-Antunano, M. A., Rodriguez-Resendiz, J., Segura, L. R., Perez, M. A. C., Corro, J. A. A., Paredes-Garcia, W. J., & Rodriguez-Resendiz, H. (2021). Analysis of emergency remote education in COVID-19 crisis focused on the perception of the teachers. *Sustainability,**13*, 3820. 10.3390/su14031121

[CR77] Zeichner, K. M. (2014). The struggle for the soul of teaching and teacher education in the USA. *Journal of Education for Teaching: International Research and Pedagogy,**40*(5), 551–568. 10.1080/02607476.2014.956544

[CR78] Zembylas, M., & Papanastasiou, E. C. (2004). Job satisfaction among school teachers in Cyprus. *Journal of Educational Administration,**42*(3), 357–374. 10.1108/09578230410534676

